# stHGC: a self-supervised graph representation learning for spatial domain recognition with hybrid graph and spatial regularization

**DOI:** 10.1093/bib/bbae666

**Published:** 2024-12-22

**Authors:** Runqing Wang, Qiguo Dai, Xiaodong Duan, Quan Zou

**Affiliations:** College of Computer Science and Engineering, Dalian Minzu University, 116600 Dalian, China; SEAC Key Laboratory of Big Data Applied Technology, Dalian Minzu University, 116600 Dalian, China; College of Computer Science and Engineering, Dalian Minzu University, 116600 Dalian, China; SEAC Key Laboratory of Big Data Applied Technology, Dalian Minzu University, 116600 Dalian, China; College of Computer Science and Engineering, Dalian Minzu University, 116600 Dalian, China; SEAC Key Laboratory of Big Data Applied Technology, Dalian Minzu University, 116600 Dalian, China; Institute of Fundamental and Frontier Sciences, University of Electronic Science and Technology of China, 611730 Chengdu, China

**Keywords:** spatial transcriptomics, spatial domain identification, self-supervised graph representation learning, hybrid neighbor graph, spatial regularization

## Abstract

Advancements in spatial transcriptomics (ST) technology have enabled the analysis of gene expression while preserving cellular spatial information, greatly enhancing our understanding of cellular interactions within tissues. Accurate identification of spatial domains is crucial for comprehending tissue organization. However, the effective integration of spatial location and gene expression still faces significant challenges. To address this challenge, we propose a novel self-supervised graph representation learning framework named stHGC for identifying spatial domains. Firstly, a hybrid neighbor graph is constructed by integrating different similarity metrics to represent spatial proximity and high-dimensional gene expression features. Secondly, a self-supervised graph representation learning framework is introduced to learn the representation of spots in ST data. Within this framework, the graph attention mechanism is utilized to characterize relationships between adjacent spots, and the self-supervised method ensures distinct representations for non-neighboring spots. Lastly, a spatial regularization constraint is employed to enable the model to retain the structural information of spatial neighbors. Experimental results demonstrate that stHGC outperforms state-of-the-art methods in identifying spatial domains across ST datasets with different resolutions. Furthermore, stHGC has been proven to be beneficial for downstream tasks such as denoising and trajectory inference, showcasing its scalability in handling ST data.

## Introduction

Spatial transcriptomics (ST) technologies, such as 10x Visium [1], Stereo-seq [2], and Slide-seqV2 [3], have revolutionized our understanding of cellular interactions within tissues by enabling the simultaneous analysis of gene expression and cellular spatial information [4]. This advancement has greatly enhanced our ability to unravel the complex organizational functions of biological systems [5]. The accurate identification of spatial domains, which involves segmenting tissue regions based on distinct gene expression patterns and spatial locations [6], is crucial for exploring biological tissue development, uncovering disease mechanisms, and guiding drug developments [7–9].

A wide range of methods have been developed for spatial domain identification, ranging from traditional clustering algorithms to advanced deep learning techniques. Traditional approaches, such as K-means and Louvain algorithms [10], often overlook spatial information, while probabilistic models like Giotto [11] and BayesSpace [12] incorporate spatial relationships but may still be limited in their ability to capture the full complexity of ST data.

More recently, deep learning methods, particularly those leveraging graph neural networks (GNNs), have shown promise in integrating spatial locations with histological features to identify spatial domains [13, 14]. Researchers have developed multiple methods for spatial domain identification, such as stLearn [15], SpaGCN [16], and DeepST [17]. These methods leverage GNNs to integrate spatial locations with histological features, grouping spots with similar gene expression patterns. However, the utility of these methods is profoundly dependent on the quality of histological imagery [18]. In contrast, utilizing only ST data offers a unique advantage in capturing gene expression characteristics, as it is unaffected by issues of image quality [19]. For instance, STAGATE [20] uses a graph attention auto-encoder (GATE) to integrate spatial information with gene expression data. This method learns complex latent representations of cells and identifies diverse spatial domains, thereby achieving better spatial domain identification. On the other hand, SEDR [19] gains deeper insights into cellular interactions and tissue organization by integrating similar features and combining a deep autoencoder with masked self-supervised learning. Additionally, GraphST [21] integrates graph convolutional networks with contrastive learning to learn embedding representations. By utilizing graph structures and traversal strategies, it concurrently elucidates interactions between local spots and highlights the structural characteristics of the entire sample. This approach optimizes the identification of spatial domains. However, despite significant progress in the field of spatial domain identification in recent years, several challenging problems remain. Firstly, the construction of neighbor graphs is crucial but often relies on a single similarity measure, making graphs sensitive to noise and outliers. Secondly, GNN-based methods can overly focus on local neighbor information, neglecting broader global relationships and leading to oversmoothing issues. Thirdly, when handling high-dimensional ST data, the model may overfit certain patterns such as local and rare gene expression patterns, leading to insufficient generalization ability.

To address these challenges, we propose a novel self-supervised graph representation learning framework based on a hybrid neighbor graph and spatial regularization, named stHGC. Firstly, a hybrid neighbor graph is constructed using multiple similarity metrics, including Euclidean distance and cosine similarity. This hybrid neighbor graph approach alleviates reliance on a single measure and reduces the impact of noise and outliers. Secondly, a graph attention auto-encoder (GATE) grounded in self-supervised contrastive learning is introduced. Within the framework, the attention mechanism captures relationships between spots in ST data, while self-supervised contrastive learning maintains the similarity of features between neighboring spots and preserves differentiation between distant spots, effectively mitigating the issue of oversmoothing. Finally, we introduce a spatial regularization constraint based on comparing neighboring and non-neighboring spots, enabling the model to better understand the continuity of spatial information and reducing the risk of overfitting. The proposed stHGC approach has been validated on multiple ST datasets from different species and spatial resolution platforms, yielding reliable outcomes in spatial domain identification. Furthermore, it has demonstrated its capabilities in downstream tasks, such as denoising and trajectory inference.

## Materials and methods

### The overview of the stHGC

**Figure 1:**
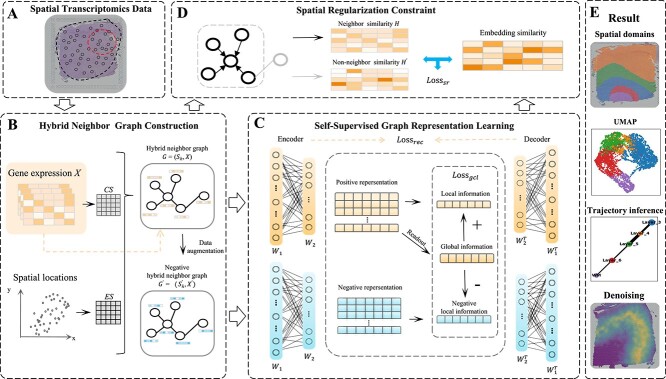
Overview of stHGC. (A) ST data includes gene expression information and spatial information for each spot. (B) The hybrid neighbor graph is constructed based on the Euclidean distance similarity matrix $ES$ calculated with a Gaussian kernel and the gene expression cosine similarity matrix $CS$, with negative spots information generated by randomly perturbing gene expression vectors. (C) The self-supervised graph representation learning framework is used to obtain low-dimensional latent representations. The weights between neighboring spots are calculated by GATE, using positive spots as self-supervised signals, and self-supervised contrastive learning compares the similarities between different spots. (D) The spatial regularization constraint is utilized to minimize the distance between spatial neighbors, maintaining the continuity of spatial information. (E) The learned representations are utilized for spatial domain identification and downstream tasks, including UMAP, trajectory inference, and denoising.

In this section, the stHGC method for identifying spatial domains from ST data is explained in detail, with its overall framework illustrated in Fig. 1. Firstly, a neighbor graph is constructed using hybrid similarity measures to reduce the reliance on a single similarity metric. Secondly, the self-supervised graph representation learning framework is proposed. stHGC employs an attention mechanism to adaptively adjust the connection weights between spots and utilizing self-supervised contrastive learning to compare the features of different spots. Thirdly, spatial regularization constraint is introduced to maintain the spatial continuity and differentiation in the representation process by making the embeddings of neighboring spots more similar and those of non-neighboring spots more distinct. Finally, a clustering algorithm is employed to group spots with similar latent representations, with each group considered a spatial domain.

### Hybrid neighbor graph construction

In ST data, the relationship between spots is related not only to spatial distance but also to gene expression at each spot [18]. Different spots may be spatially proximal but exhibit completely different gene expression patterns. To comprehensively represent the relationships between spots, we propose constructing a hybrid neighbor graph using both of similarity measures to capture both spatial proximity and similarity in high-dimensional feature space among spots. It is designed to deliver a more comprehensive description of the relationships between spots in ST data. Prior to constructing the hybrid neighbor graph, data preprocessing is essential. Initially, the top $M$ highly variable genes are selected (in this study, $M$ was set to 3000). Subsequently, the retained gene expression data are normalized to ensure comparability across different spots. Lastly, a logarithmic transformation is applied to further diminish the disparities between gene expression values.

Let $X=\{x_{1},x_{2},x_{3},...,x_{N}\}\in \mathbb{R}^{N \times M}$ be the gene expression matrix, where $N$ denotes the total number of spots, and $x_{i}$ represents the gene expression feature of the $i$th spot. Let $C=\{(a_{1},b_{1}),(a_{2},b_{2}),...,(a_{N},b_{N})\}\in \mathbb{R}^{N \times 2}$ be the coordinates of spots, where $(a_{i},b_{i})$ is the coordinate of the $i$th spot.

Construct the hybrid neighbor graph as follows:

(1) All spots with Euclidean distances within the pre-defined radius $d$ are considered neighbors in the subsequent calculation of the adjacency matrix.
(2) The spatial proximity between spots $i$ and $j$ is determined by calculating the Euclidean distance within a similarity matrix that utilizes the Gaussian kernel function:
  (1) \begin{align*}& ES_{ij} = \exp\left(-\frac{\|C_{i} - C_{j}\|^{2}}{2\eta^{2}}\right)\end{align*}
  where $C_{i}$ and $C_{j}$ are the coordinates of spots $i$ and $j$, respectively, and $\eta $ is the standard deviation of the Gaussian kernel.
(3) When dealing with high-dimensional data such as gene expression, Euclidean distance may be sensitive to small changes in spatial position [22]. In contrast, cosine similarity can capture similarities in gene expression patterns and is not influenced by feature dimensions and scales [18]. Therefore, cosine similarity is used to calculate the similarity between spot pairs:
  (2) \begin{align*}& CS_{ij} = \frac{x_{i} \cdot x_{j}}{\|x_{i}\|\|x_{j}\|}\end{align*}
  where $ x_{i} $ and $ x_{j} $ are the gene expression vectors of spots $ i $ and $ j $, respectively.
(4) A weighted approach is employed to construct the spatial adjacency matrix $S_{h}$:
  (3) \begin{align*}& {S_{h}}_{ij} = \alpha ES_{ij} + \beta CS_{ij}\end{align*}
  where $\alpha $ and $\beta $ are the weights of the two adjacency matrices, respectively. In $S_{h}$, neighbors are considered if the similarity between two spots is greater than 0. This ensures that only neighbors with significant spatial and gene expression associations are preserved.
(5) The neighbor relationships are constructed into a hybrid neighbor graph $G = (S_{h}, X)$, which takes into account both spatial distance and gene expression features. This hybrid neighbor graph not only considers the spatial adjacency between spots but also takes into account the similarity of their gene expression features, thus more effectively capturing the complex relationships between spots in ST data.

### Self-supervised graph representation learning framework

We propose self-supervised graph representation learning framework for learning the representations of hybrid neighbor graphs. Initially, an attention mechanism is introduced to dynamically adjust the importance weights of neighboring spots. In addition, a self-supervised contrastive learning module is utilized to yield more discriminative feature representations of spots by comparing the similarities of local and global features.

#### Graph attention auto-encoder based on hybrid neighbor graph

To enable stHGC to delineate local characteristics and intricate spatial associations within ST data, the GATE [23] is employed. This method dynamically assigns varying importance weights to neighboring spots through an attention mechanism.

In the encoder of GATE, the normalized gene expression data are utilized as the input to the model. Let $x_{i}$ represent the normalized expression of spot $i$, and $n$ denote the number of layers in the encoder. With gene expression serving as the initial spot embeddings, the encoder layer computes the embedding for spot $i$ in the $n$th layer as follows:


(4) \begin{align*}& u_{i}^{\left( n \right)}=\sigma \left( \sum_{j \in G_{i}}{a_{ij}^{\left( n \right)}\left( T_{k}u_{j}^{\left( n-1 \right)} \right)} \right)\end{align*}

where $T_{k}$ is the trainable weight matrix, $\sigma $ is the nonlinear activation function, $G_{i}$ is the set of neighbors of spot $i$ in the hybrid neighbor graph, and $a_{ij}^{\left ( n \right )}$ is the normalized edge weight between spot $i$ and spot $j$ in the output of the $n$th attention layer. The final learned embeddings are denoted as $\mathit{U}$.

The output from the encoder of GATE is input into the decoder to reconstruct the gene expression, the embedding of spot $i$ in $(n-1)$th layer is reconstructed by the decoder layer in $n$th layer, as follows:


(5) \begin{align*}& \hat{u}_{i}^{(n-1)} = \sigma\left(\sum_{j \in G_{i}}{a_{ij}^{(n-1)}(T_{ij}\hat{u}_{j}^{(n)})}\right)\end{align*}

In both the encoder and decoder of GATE, a single-layer feed-forward neural network attention mechanism is introduced to parameterize the relationships between spots based on the calculated weights. In the $n$th layer, the edge weights from spot $i$ to its neighboring spot $j$ are computed and normalized using the activation function:


(6) \begin{align*}& a_{ij}^{\left( n \right)}=\frac{\exp \left( \phi\left( \delta_{s}\left( T_{k}u_{i}^{\left( n-1 \right)} \right) +\delta_{r}\left( T_{k}u_{j}^{\left( n-1 \right)} \right) \right) \right)}{\sum_{\substack{1 \leq i \leq N}}{\exp \left( \phi\left( \delta_{s}\left( T_{k}u_{i}^{\left( n-1 \right)} \right) +\delta_{r}\left( T_{k}u_{j}^{\left( n-1 \right)} \right) \right) \right)}}\end{align*}

where $\delta _{s}$, $\delta _{r}$, and $T_{k}$ are trainable weight vectors, $\phi $ is the activation function, and $X$ is the gene expression of spot $i$. $u_{i}^{(n-1)}$ and $u_{j}^{(n-1)}$ represent the feature representations of spot $i$ and spot $j$ in the $(n-1)$th layer, respectively.

The reconstruction loss for GATE is defined by minimizing the error between the encoder’s input and the decoder’s output, as follows:


(7) \begin{align*}& L_{rec}=\sum_{i=1}^{N}{\lVert x_{i}-\hat{u}_{i} \rVert^{2}}\end{align*}

By minimizing this loss, the model can learn latent representations of the input spots.

#### Self-supervised contrastive learning

To enhance the discriminative capacity of the representations, we adopted a self-supervised contrastive learning strategy. In this process, data augmentation is a critical step. First, in the gene expression data, positive spots are constructed based on the hybrid neighbor graph $G = (S_{h}, X)$. Second, to generate negative spots that offer a different view from the positive spots, we construct them by randomly shuffling the rows of the gene expression matrix $X$ (i.e. shuffling the spots), resulting in $X^{\prime}$. Finally, a negative sample hybrid neighbor graph $G^{\prime} = (S_{h}^{\prime}, X^{\prime})$ is constructed, where $X^{\prime}$ and $G^{\prime}$ denote the shuffled gene expression matrix and the negative sample hybrid neighbor graph, respectively.

In ST data, the gene expression of individual spots within the same tissue slice typically is related to the global characteristics of that tissue slice [24]. This inherent coherence acts as a supervising signal, which can be utilized to guide the learning process of spot embeddings.

Specifically, inspired by the DGI [25] a graph-level summary representation is generated through the readout function $R: \mathbb{R}^{N \times M} \rightarrow \mathbb{R}^{M}$, which is denoted as ${\mathbf{r}} = R(\mathit{U})$. Here, $\mathit{U}$ is used to represent the embeddings of spots, and $\mathbf{r}$ is the global graph-level summary. Then, the discriminator $D$ employs a bilinear layer to assess the similarity between positive and negative samples in relation to the global summary representation of the graph. Finally, a binary cross-entropy loss function as follows:


(8) \begin{align*}& L_{gcl} = -\frac{1}{2N} \left( \sum_{i=1}^{N} \left( \mathbb{E}_{(X, \mathit{U})} \left[ \log D(\mathit{U}, \mathbf{r}) \right] + \mathbb{E}_{(X^{\prime}, U^{\prime})} \left[ \log \left( 1 - D(\mathit{U^{\prime}}, \mathbf{r}) \right) \right] \right) \right)\end{align*}

where $\mathit{U^{\prime}}$ represents the embeddings of the corresponding spots in the negative sample graph $G^{\prime}$.

### Spatial regularization

A spatial regularization constraint is introduced to better utilize spatial information, which aids in the reduction of overfitting risks. This method enhances data representation by considering the spatial proximity between spots, thereby improving the understanding of spatial patterns in ST data.

Specifically, a loss function for spatial regularization constraint is defined to measure the similarity of expression features between spatially neighboring and non-neighboring spots as follows:


(9) \begin{align*}& L_{sr}=-\sum_{i=1}^{N}{\left( \sum_{j \in N_{i}}{\log \left( \psi \left( H_{ij} \right) \right) + \sum_{o \notin N_{i}}{\log \left( 1-\psi \left( H^{\prime}_{io} \right) \right)}} \right)}\end{align*}

where $\psi $ represents the activation function, $H_{ij}$ represents the cosine similarity between spot $i$ and its neighboring spot $j$, $H^{\prime}_{io}$ represents the cosine similarity between spot $i$ and its non-neighboring spot $o$, and $N_{i}$ is the set of neighboring spots for spot $i$.

### Model optimization

In the training process of stHGC, the aforementioned three types of loss functions, namely $L_{rec}$, $L_{gcl}$, and $L_{sr}$, are jointly optimized. This joint optimization serves as the final training objective as follows:


(10) \begin{align*}& L=\lambda L_{rec}+\mu L_{gcl}+\gamma L_{sr}\end{align*}

where $\lambda $, $\mu $, and $\gamma $ are the weights of different loss functions, and are determined by experimental tuning.

The stHGC embeddings $\mathit{U}$ are utilized to identify spatial domains. Spots are clustered into different clusters using the mclust [26] method, with each identified cluster representing a spatial domain.

## Results

### Datasets

To assess the performance of the stHGC model, we employed a diverse set of ST datasets that are generated from several platforms with different resolutions. Specifically, as depicted in Table 1, these datasets included one centered on the human dorsolateral prefrontal cortex (DLPFC) dataset, produced using 10x Visium technology and comprising 12 slices [6]; the mouse olfactory bulb dataset obtained via Stereo-seq [19]; another mouse olfactory bulb dataset acquired by Slide-seqV2 [27]; and datasets of human breast cancer and bronchiolar adenoma obtained through the 10x Visium platform [2]. These datasets, characterized by their intricate compositional nature, have been widely employed in research endeavors across diverse scientific teams [21, 24, 28], serving as a stringent benchmark to gauge the efficacy of stHGC.

**Table 1:** Statistical information on all datasets used in the study

Datasets	Organisms	Slices	Spots	Genes	Domains	Platforms
DLPFC	Human	12	3460–3789	33 538	5–7	10x Visium
Olfactory bulb	Mouse	1	19 109	14 376	7	Stereo-seq
Olfactory bulb	Mouse	1	20 139	18 675	9	Slide-seqV2
Breast cancer	Human	1	3798	36 601	20	10x Visium
Bronchiolar adenoma	Human	1	4002	36 601	4	10x Visium

### Evaluation metrics

The Adjusted Rand Index (ARI) [29] and Normalized Mutual Information (NMI) [30] are utilized in this work as evaluation metrics. These metrics are derived by contrasting the clustered labels produced by the tested method against the true labels of each sample, evaluating their agreement to quantify the similarity between the two sets of labels. The ARI, a chance-adjusted Rand index, provides a more reliable measure of clustering accuracy, ranging from -1 to 1, with higher values signifying better clustering performance. NMI is based on mutual information, which quantifies the shared information between two data distributions, effectively evaluating both overlapping and non-overlapping clustering. The range of NMI values is 0 to 1, with higher values reflecting better clustering outcomes.

For datasets with unknown true labels, we use the Davies–Bouldin Index (DB) [31] and Cophenetic Correlation Coefficient (CCC) [32] to evaluate clustering performance. DB assesses the compactness and separation of clusters by measuring the similarity ratio between each cluster and its most similar counterpart. Lower DB values indicate that spots within clusters are more tightly grouped, while the separation between different clusters is higher. CCC reflects the stability and validity of the clustering, with values ranging from 0 to 1. A higher CCC value indicates that the clustering results better preserve the original structure of the samples, suggesting that the model captures the data structure more accurately. The Jaccard index [33] is used to measure the overlap between the expression of marker genes in a specific domain and the overall gene expression within that domain. A higher Jaccard index indicates a greater overlap, implying better clustering performance.

Together, these metrics offer diverse viewpoints on clustering performance, providing a more comprehensive evaluation of the clustering results.

### stHGC improves the performance of identifying known layers on the DLPFC dataset

The performance of the proposed method for identifying spatial domains in twelve slices of the DLPFC [6] was validated. The DLPFC datasets were manually annotated with authentic labels, including DLPFC layers (4-6 layers) and white matter (WM) [6]. Seven existing methods were compared, including one non-spatial method (implemented via SCANPY [34]), three methods based on ST data (STAGATE [20], GraphST [21], SEDR [19]), and three methods incorporating image features (stLearn [15], DeepST [17], conST [35]). All methods were run with their default parameters on the same dataset.

**Figure 2:**
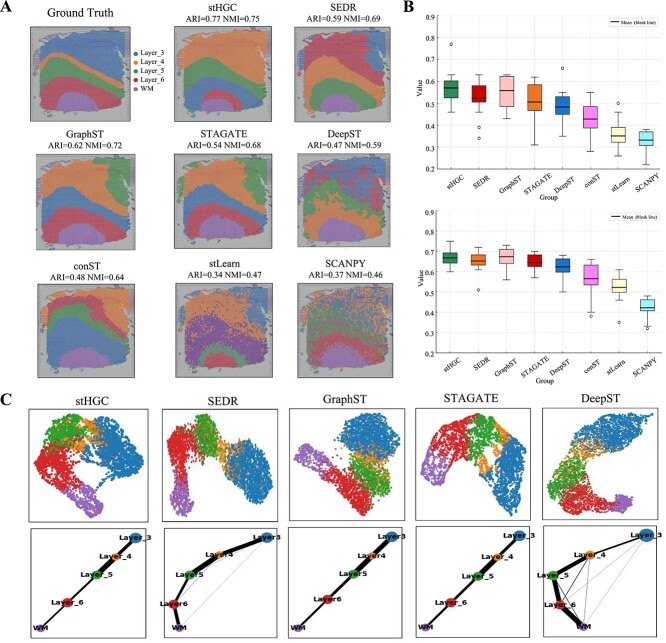
Comparison of spatial domain identification results between stHGC and seven other methods on the human DLPFC dataset. (A) Clustering results of this work’s method with seven other methods (SEDR, GraphST, STAGATE, DeepST, conST, stLearn, SCANPY) on 151 672 slices. Along with manually annotated real clustering results. (B) Distribution of ARI and NMI values for 12 slices of DLPFC on stHGC and other methods. (C) Results of UMAP and PAGA plots generated by the five methods, stHGC, SEDR, GraphST, STAGATE, and DeepST.

As shown in 2, the stHGC method effectively recognized each layer domain on the twelve DLPFC slices, outperforming other methods. For example, in sample 151672, the distribution of all layers was effectively detected by stHGC, achieving high clustering accuracy (ARI=0.77, NMI=0.75) ( Fig. 2A). The analysis revealed that, with the exception of the SCANPY approach, all other methods identified a five-layer structure, but some aspects still showed deficiencies. The stLearn and conST methods exhibited discontinuous cluster boundaries. DeepST also showed insufficient clustering accuracy, with unclear layer boundaries. SEDR, GraphST, and STAGATE incorrectly clustered layer_3 into two parts and unable to effectively cluster layer_4. These experimental results demonstrate that stHGC identifies spatial domains in ST data more clearly and highlight the necessity of utilizing spatial information.

Figure 2(B) showed the box plot distribution of ARI and NMI values across the 12 slices, demonstrating the effectiveness of these methods on the DLPFC dataset. Clearly, the stHGC method exhibited excellent performance in terms of mean evaluation results (ARI=0.57, NMI=0.67), indicating that the model’s performance was more centralized across the 12 slices, with less influence from outliers. The ARI values for SCANPY and stLearn were generally low. The evaluation results for conST, DeepST, STAGATE, and SEDR were widely distributed. While GraphST’s evaluation results were relatively stable, stHGC showed slightly better consistency.

Combining gene expression with spatial information enabled the stHGC method to achieve gene expression embedding and spatial trajectory inference (Fig. 2C), which could explore whether the development of individual cortical layers was organized [36]. For example, in Fig. 2(C), the UMAP [37] plot generated by stHGC showed well-organized cortical layers. Additionally, the trajectory inference generated by the stHGC method using the PAGA [38] technique depicted a linear trajectory from layer_3 to the WM region. In contrast, the trajectory inferences of SEDR and DeepST were not linear. The inferred trajectory of GraphST was linear; however, the separation of spots from different layers in the UMAP plot was not distinct. Although STAGATE exhibited clearer separation between different layers in the UMAP plot, stHGC also demonstrated the ability to depict a linear trajectory similar to that of STAGATE in trajectory inference. These results validated that stHGC improved spatial domain identification performance across multiple consecutive DLPFC datasets while contributing to the preservation of their underlying biological features.

These results validated that stHGC improved spatial domain identification performance across multiple consecutive DLPFC datasets while contributing to the preservation of their underlying biological features.

### Spatial domain identification across different spatial resolution platforms using stHGC

In this subsection, we evaluated the performance of the stHGC method on mouse olfactory bulb datasets generated by Stereo-seq [39] and Slide-seqV2 [40]. For comparative analysis, four additional methods were selected, including three spatial clustering approaches—SEDR, GraphST, and STAGATE—as well as a non-spatial clustering approach implemented by SCANPY.

**Figure 3:**
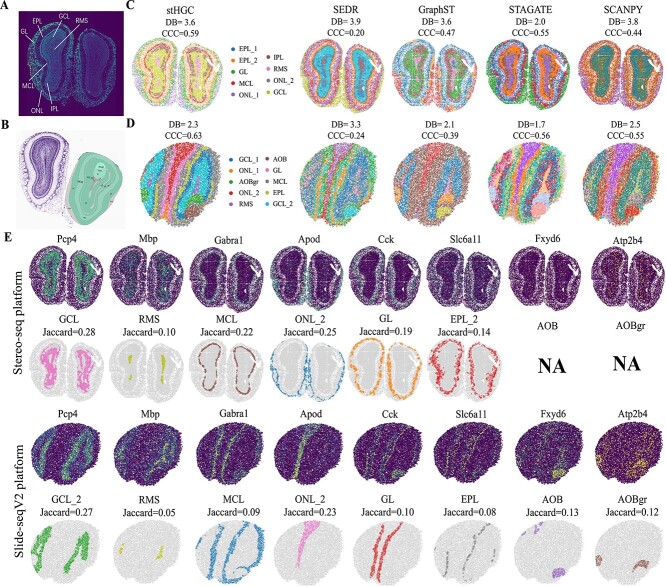
Application of the stHGC method to mouse olfactory bulb tissues on the Stereo-seq and Slide-seqV2 platforms. (A) Annotated map of the laminar organization of the mouse olfactory bulb on DAPI-stained images. (B) Images of the mouse olfactory bulb from the Allen Mouse Brain Atlas. (C) Spatial domain clustering results of mouse olfactory bulb datasets on the Stereo-seq platform obtained by applying five methods: stHGC, SEDR, GraphST, STAGATE, and SCANPY. (D) Spatial domain clustering results of mouse olfactory bulb datasets on the Slide-seq platform obtained by applying the same five methods. (E) stHGC identifies the spatial domains and corresponding marker genes in the slices from both platforms (Stereo-seq and Slide-seqV2). NA indicates unrecognized spatial domains.

The Stereo-seq platform, which utilized DNB chips and *in situ* RNA capture technology, offered wide-field nanoscale resolution for spatiotemporal omics [39]. The DAPI-stained images annotated the coronal mouse olfactory bulb’s layered structure: the olfactory nerve layer (ONL), the rostral migratory stream (RMS), the external plexiform layer (EPL), the internal plexiform layer (IPL), the glomerular layer (GL), the mitral cell layer (MCL), and the granule cell layer (GCL). While the DB score for stHGC (DB=3.6) was lower compared to STAGATE (DB=2.0), it demonstrated better performance in the CCC metric (CCC=0.59) (Fig. 3C). This was further validated by comparing the known marker gene expression patterns[41] with the visualizations of each spatial domain from stHGC and four other methods (SEDR, GraphST, STAGATE, and SCANPY) (Fig. 3E, Supplementary Figure 5). stHGC delineated the clustering boundaries of each domain more distinctly, especially for the GL, GCL, and EPL domains. SEDR incorrectly identified the MCL domain when detecting the RMS domain. GraphST did not consistently identify the MCL domain, while STAGATE failed to clearly define the boundary between the GCL and RMS domains. The non-spatial clustering method implemented by SCANPY failed to identify the GL and EPL domains. From Supplementary Table 1, STAGATE achieved the highest Jaccard index in the RMS and ONL domains (0.11, 0.34), while stHGC outperformed other methods in the MCL, GL, and EPL domains (0.22, 0.19, 0.14). This indicates a notable competitive advantage of stHGC in spatial domain identification.

Additionally, stHGC was applied to the Slide-seqV2 [40] profiled mouse olfactory bulb ST dataset, which featured a 10 $\mu $m spatial resolution. In addition to the spatial domains included in the Stereo-seq platform dataset, the dataset generated by Slide-seqV2 also contained two specific spatial structures, namely the accessory olfactory bulb (AOB) and the granular layer of the accessory olfactory bulb (AOBgr), which might be due to differences in sampling locations [28]. Fxyd6 and Atp2b4 were respectively used to validate AOB and AOBgr [28]. By comparing with the mouse olfactory bulb atlas from the Allen Mouse Brain Atlas (Fig. 3B) [42], it was found that stHGC not only clustered the same six spatial domains as those identified in the Stereo-seq platform dataset but also effectively identified specific regions (AOB and AOBgr) in the Slide-seqV2 platform dataset. Fig. 3(D) shows a comparison between stHGC and four other methods: both stHGC and STAGATE clearly identify the MCL domain, but stHGC delineates the boundaries between different spatial domains more distinctly. The non-spatial clustering method SCANPY shows unclear boundaries between adjacent domains. GraphST incorrectly clusters the MCL, EPL, and GL as a single spatial domain. SEDR fails to identify the AOBgr domain. As shown in Fig. 3(D), stHGC (DB=2.3) displayed a slightly inferior performance in the DB metric compared to STAGATE (DB=1.7) and GraphST (DB=2.1). However, it outperformed all other tested methods in the CCC metric (CCC=0.63). This demonstrates that stHGC has competitive performance in distinguishing between different spatial domains. Similarly, on this dataset, the effectiveness of these five methods in identifying spatial domains was further validated by comparing the visualization results of each spatial domain with its corresponding known marker gene expression patterns (Fig. 3(E), Supplementary Figure 6). From Supplementary Table 2, it can be seen that stHGC outperforms other methods in terms of Jaccard index in the MCL, EPL, AOB, and AOBgr (0.09, 0.08, 0.13, 0.12). STAGATE achieved excellent indices in the GCL and AOB domains (0.44, 0.13). Although other methods achieved good metrics in certain spatial domains, there are still some spatial domains that remain unidentified.

In general, the testing results indicated that stHGC effectively mitigated overfitting issues, enhanced the model’s generalization ability, and improved the identification of spatial domains in ST data across different spatial resolutions.

### Performance of the stHGC method in identifying spatially heterogeneous structures in human cancer tissue

**Figure 4:**
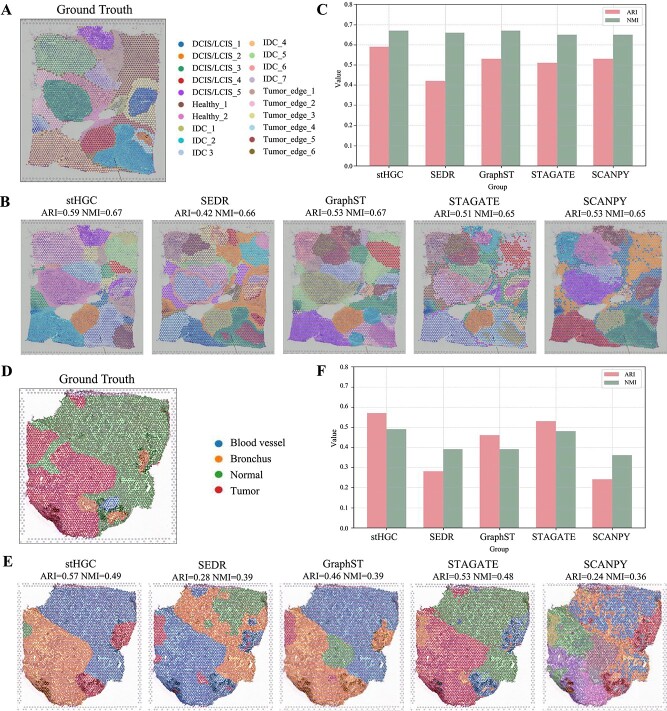
Spatial domain identification by applying stHGC to two cancer datasets (HBC and BA). (A) Manually annotated human breast cancer (HBC) dataset. (B) Spatial domain identification results of the HBC dataset using five methods (stHGC, SEDR, GraphST, STAGATE, SCANPY). (C) Histograms of ARI and NMI assessment values for the HBC dataset obtained by applying the five methods. (D) Manually annotated fine BA dataset. (E) Spatial domain identification results of the BA dataset using five methods (stHGC, SEDR, GraphST, STAGATE, SCANPY). (F) Histograms of ARI and NMI assessment values for the BA dataset obtained by applying the five methods.

Due to the complexity of the microstructure of cancer tissues, we applied stHGC and four other methods to cancer tissue datasets to further validate their performance in identifying heterogeneous spatial structures in cancer tissues. We selected the human breast cancer (HBC) tissue dataset [43] and the human bronchial adenoma (BA) tissue dataset [24], which were generated by the 10x Visium platform, The HBC dataset is annotated to include normal healthy tissue (Healthy), invasive ductal carcinoma (IDC), ductal carcinoma *in situ*/lobular carcinoma *in situ* (DCIS/LCIS), and peri-tumor regions with fewer malignant features [43] (Fig. 4A). As shown in Fig. 4(B), stHGC is more consistent with manual annotations in spatial domain identification and can more effectively and smoothly label multiple specific tumor regions compared to the other four methods (SEDR, GraphST, STAGATE, SCANPY). SCANPY and STAGATE fail to continuously identify IDC and Tumor_edge domains, leading to confusion between identified domains and blurred boundaries of neighboring clusters. SEDR and GraphST can identify spatial domains more continuously, but their identification of the IDC domain shows significant discrepancies compared to manual annotations. The bar charts of ARI and NMI evaluation values reveal that stHGC achieved the highest values for both results (ARI=0.59, NMI=0.67) (Fig. 4C).

Bronchial adenoma (BA) is an infrequent lung tumor typically arising in the bronchial epithelium, with ST analysis of these tumors being rarely conducted [44]. The BA dataset includes different regions [24], such as Tumor, Normal, Bronchus, and Blood vessel (Fig. 4D). As shown in Fig. 4(E), the stHGC method outperforms other methods in recognizing spatial domains, especially in the Tumor and Normal domains. SCANPY incorrectly identified 10 domains, while SEDR was unable to identify the Normal domain continuously. GraphST and STAGATE were unable to identify continuous tumor domains. Figure 4(F) presents the qualitative and quantitative analyses of these four methods on the BA dataset.

The analysis results in this section indicate that utilizing stHGC to analyze ST data from different cancer datasets can uncover regional gene expression variations in cancer tissues, offering deeper insights into the spatial heterogeneity of tumors.

### Validation of stHGC for denoising of gene expression

Spatial transcriptome data are often noisy, and some data may dropout during the profiling process, potentially affecting the accuracy of gene expression analysis [18].

**Figure 5:**
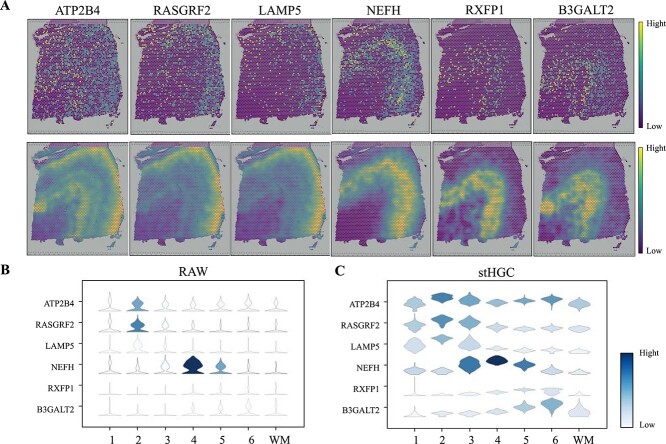
stHGC was utilized to remove noise and enhance the expression of layer-marked genes in DLPFC sections. (A) The raw expression of six layer-marker genes on section 151673 from the DLPFC dataset (upper) and the visualization of gene expression after denoising using the stHGC method (lower). (B) Violin plot of the raw expression of layer-marker genes. (C) Violin plot of gene expression after denoising.

stHGC was validated on DLPFC’s 151673 slices to reveal the spatial expression patterns of genes, demonstrating efficient denoising capabilities in this task (Fig. 5A). Six layer-marker genes (ATP2B4, RASGRF2, LAMP5, NEFH, RXFP1, and B3GALT2) [45] were selected to compare their expression levels in the original dataset with those in the datasets denoised by stHGC and three other methods (SEDR, GraphST, and STAGATE) (Fig. 5, Supplementary Figure 7). Specifically, Fig. 5(A) and Supplementary Figure 7 illustrate the spatial expression of layer-marker genes after data enhancement. In the original data, genes like LAMP5 and RXFP1 exhibit sparse expression patterns, making it challenging to discern a clear pattern (Fig. 5A). stHGC and the other three methods showed good performance in denoising and enhancing gene expression (Supplementary Figure 7A). However, SEDR’s performance on genes like RXFP1 and B3GALT2 showed blurred boundaries in the WM and layer_6. Compared to GraphST, stHGC and STAGATE retained a smoother expression distribution while preserving the gene expression pattern structure. For NEFH, stHGC demonstrates clearer spatial boundaries between the white matter (WM) and layer_6 compared to STAGATE.

Additionally, violin plots were used to compare the raw and estimated expression data from the four aforementioned methods (Fig. 5B, Supplementary Figure 7B). This allows for observing the distribution shape of each gene in different groups, and to determine whether the grouping differences are significant and whether the distribution is reasonable. In the figure, stHGC and STAGATE showed clearer differences in gene expression, with more distinct groupings, while the original data distribution appeared more scattered. In comparison, GraphST and SEDR seemed to be slightly less effective than stHGC in reflecting differences in distribution. For example, NEFH expression showed no significant difference between layer_5 and layer_6 in GraphST results, and ATP2B4 did not exhibit clear differences in distribution in SEDR’s enhancement results.

These results indicated that the gene expression data reconstructed by the stHGC method reduced noise interference, allowing clearer observation of the expression patterns of layer-marker genes across domains. This enhanced our understanding of subtle gene expression differences across different regions of the brain.

### Ablation study

**Figure 6:**
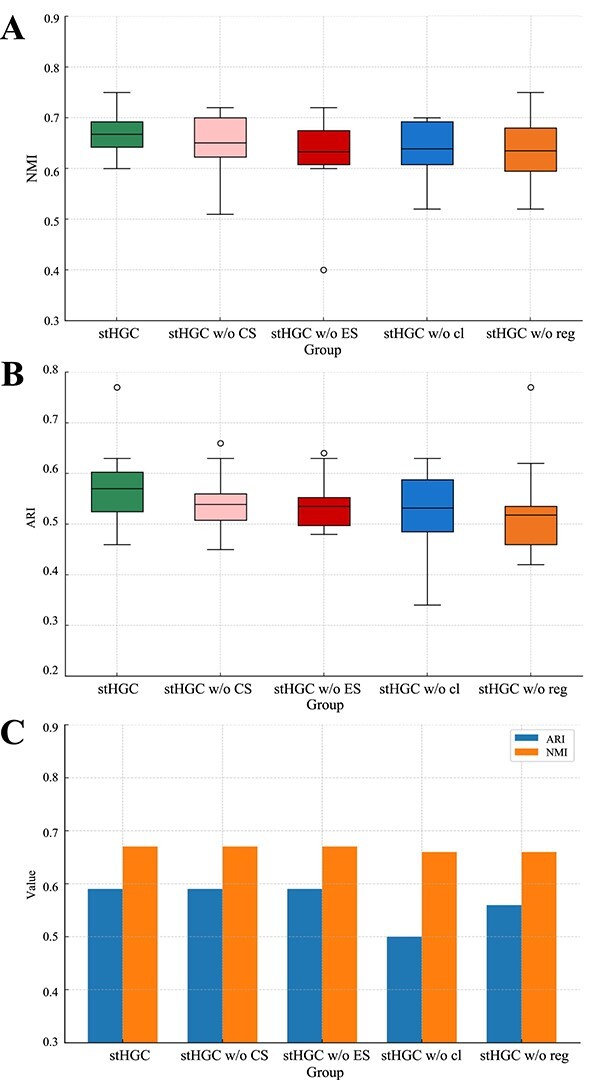
stHGC and its four variants were applied to the DLPFC and HBC datasets. (A) NMI evaluation metric for the DLPFC dataset. (B) ARI evaluation metric for the DLPFC dataset. (C) ARI and NMI evaluation metrics for the HBC dataset.

The previous section demonstrated the reliable performance of the stHGC method compared to other methods. In this subsection, we conducted ablation experiments and designed four stHGC variants. The first two variants used only Euclidean distance to construct the neighbor graph (stHGC w/o CS) and only cosine similarity to construct the neighbor graph (stHGC w/o ES), respectively. The third variant did not use self-supervised contrastive learning (stHGC w/o cl), and the fourth variant did not use spatial regularization (stHGC w/o reg). These variants were used to assess the impact of each component on the overall model performance. Figure 6 clearly showed the distribution of ARI and NMI values for the four variants and the stHGC method across 12 DLPFC slices and the HBC slice.

As shown in Fig. 6(A, B), on the DLPFC dataset, the ARI and NMI values for the four variants—stHGC w/o CS (mean: ARI=0.54, NMI=0.65), stHGC w/o ES (mean: ARI=0.54, NMI=0.63), stHGC w/o cl (mean: ARI=0.53, NMI=0.65), and stHGC w/o reg (mean: ARI=0.52, NMI=0.64)—were all inferior to those of stHGC (mean: ARI=0.57, NMI=0.67). This corroborates the significance of employing hybrid neighbor graph, self-supervised contrastive learning, and spatial regularization in discerning datasets with relatively few spatial domains. Figure 6(C) demonstrates that for the HBC dataset, which has a large number of spatial domains, both stHGC w/o cl (ARI=0.50, NMI=0.66) and stHGC w/o reg (ARI=0.56, NMI=0.66) underperform relative to stHGC(ARI=0.59, NMI=0.67), underscoring the efficacy of self-supervised contrastive learning and spatial regularization in identifying multiple spatial domains. However, the variants stHGC w/o CS (ARI=0.59, NMI=0.67) and stHGC w/o ES (ARI=0.59, NMI=0.67) show no significant difference in performance compared to stHGC (ARI=0.59, NMI=0.67) on the HBC dataset. This might have been due to the higher complexity of neighborhood relationships in the HBC dataset, which obscured or smoothed between similarity measures.

Through these ablation experiments, it could be seen that each module in the stHGC method had its own importance. The integration of self-supervised contrastive learning and spatial regularization in stHGC facilitated more effective clustering of spatial domains and enhanced the overall model performance. At the same time, the hybrid neighbor graph played an important role in handling cases with fewer spatial domains, but further exploration was needed for datasets with complex microenvironments.

## Discussion and conclusion

Accurately identifying spatial domains was essential for understanding tissue structures and biological functions in microenvironments. To this end, we proposed a self-supervised graph representation learning framework for spatial domain recognition with a hybrid neighbor graph and spatial regularization (stHGC). stHGC optimized ST data processing and analysis in several ways. Firstly, it constructed a hybrid neighbor graph by considering different similarity measures to thoroughly analyze spatial relationships from multiple perspectives. Secondly, during embedding, the self-supervised graph representation learning framework adaptively adjusted weights, reducing noise and irrelevant information. Contrastive learning further uncovered potential spatial patterns and relationships by comparing similarities between different spots. Finally, the spatial regularization constraint introduced losses for neighbors and non-neighbors, effectively reflecting spatial adjacency relationships during representation learning and optimizing the learned representations. This method was tested on five ST datasets from different spatial resolution platforms. The results showed that the spatial domains identified by stHGC were more consistent with manually annotated real spots and could extend to downstream tasks.

Despite its advantages, the stHGC’s computational complexity and resource intensity pose challenges for larger and higher-resolution datasets. The rapid advancement of ST technology offers unlimited prospects for finer measurements and richer datasets. Future optimizations may include using parallel processing and small-batch processing to tackle resource issues. Additionally, we are exploring multi-slice mapping to improve ST data analysis due to the significant connections between consecutive slices.

**Key Points**

- stHGC is a novel self-supervised graph representation learning framework that accurately identifies spatial domains, and effectively performs downstream tasks such as UMAP visualization, trajectory inference, and denoising.
- stHGC employs a hybrid neighbor graph approach with different similarity measures, facilitating a comprehensive analysis of spatial relationships from multiple perspectives.
- A spatial regularization constraint is implemented to enhance the distinction between neighboring and non-neighboring spots, ensuring continuity in spatial relationship representations.
- Experiments demonstrate that stHGC outperforms other state-of-the-art methods in the spatial domain identification tasks on ST datasets across different spatial resolutions and species.

## Supplementary Material

Supplementary_file_bbae666

## Data Availability

The code and data of the proposed framework can be accessed through the GitHub repository at:(https://github.com/DaiLab-DLNU/stHGC).

## References

[1] Rao N , Clark S, Habern O. Bridging genomics and tissue pathology: 10x genomics explores new frontiers with the visium spatial gene expression solution. Gen Eng Biotechnol News 2020; 40:50–1. 10.1089/gen.40.02.16.

[2] Wei X , Sulei F, Li H. et al. Single-cell Stereo-seq reveals induced progenitor cells involved in axolotl brain regeneration. Science 2022; 377:eabp9444. 10.1126/science.abp9444. 36048929

[3] Robert R , Stickels EM, Kumar P. et al. Highly sensitive spatial transcriptomics at near-cellular resolution with Slide-seqV2. Nat Biotechnol 2021; 39:313–9. 10.1038/s41587-020-0739-1. 33288904 PMC8606189

[4] Asp M , Bergenstråhle J, Lundeberg J. Spatially resolved transcriptomes—next generation tools for tissue exploration. Bioessays 2020; 42:e1900221. 10.1002/bies.201900221. 32363691

[5] Cheng A , Guanyu H, Li WV. Benchmarking cell-type clustering methods for spatially resolved transcriptomics data. Brief Bioinform 2023; 24:bbac475. 10.1093/bib/bbac475. 36410733 PMC9851325

[6] Maynard KR , Collado-Torres L, Weber LM. et al. Transcriptome-scale spatial gene expression in the human dorsolateral prefrontal cortex. Nat Neurosci 2021; 24:425–36. 10.1038/s41593-020-00787-0. 33558695 PMC8095368

[7] Zeng Z , Li Y, Li Y. et al. Statistical and machine learning methods for spatially resolved transcriptomics data analysis. Genome Biol 2022; 23:83. 10.1186/s13059-022-02653-7. 35337374 PMC8951701

[8] Zhang T , Zhang Z, Li L. et al. GTAD: a graph-based approach for cell spatial composition inference from integrated scRNA-seq and ST-seq data. Brief Bioinform 2024; 25:bbad469. 10.1093/bib/bbad469 PMC10734610 38127088

[9] Zhang T , Zhang Z, Li L. et al. GTADC: a graph-based method for inferring cell spatial distribution in cancer tissues. Biomolecules 2024; 14:436. 10.3390/biom14040436. 38672453 PMC11048052

[10] Blondel VD , Guillaume J-L, Lambiotte R. et al. Fast unfolding of communities in large networks. J Stat Mech: Theor Exp 2008; 2008:P10008. 10.1088/1742-5468/2008/10/P10008.

[11] Dries R , Zhu Q, Dong R. et al. GIOTTO: a toolbox for integrative analysis and visualization of spatial expression data. Genome Biol 2021; 22:78. 10.1186/s13059-021-02286-2. 33685491 PMC7938609

[12] Zhao E , Stone MR, Ren X. et al. Spatial transcriptomics at subspot resolution with bayesspace. Nat Biotechnol 2021; 39:1375–1384. 10.1038/s41587-021-00935-2 PMC8763026 34083791

[13] Fang Z , Liu T, Zheng R. et al. stAA: adversarial graph autoencoder for spatial clustering task of spatially resolved transcriptomics. Brief Bioinform 2024; 25:bbad500. 10.1093/bib/bbad500 PMC10772985 38189544

[14] Duan H , Zhang Q, Cui F. et al. MVST: identifying spatial domains of spatial transcriptomes from multiple views using multi-view graph convolutional networks. PLoS Comput Biol 2024; 20:e1012409. 10.1371/journal.pcbi.1012409 39235988 PMC11376559

[15] Pham D, Tan X, Jun X. et al. stLearn: integrating spatial location, tissue morphology and gene expression to find cell types, cellcell interactions and spatial trajectories within undissociated tissues. BioRxiv 2020. 10.1101/2020.05.31.125658.

[16] Hu J, Li X, Coleman K. et al. SpaGCN: Integrating gene expression, spatial location and histology to identify spatial domains and spatially variable genes by graph convolutional network[J]. Nature Methods, 2021; 18:1342–1351. 10.1038/s41592-021-01255-8. 34711970

[17] Chang X , Jin X, Wei S. et al. DeepST: identifying spatial domains in spatial transcriptomics by deep learning. Nucleic Acids Res 2022; 50:e131. 36250636 10.1093/nar/gkac901 PMC9825193

[18] Wang B , Luo J, Liu Y. et al. Spatial-MGCN: a novel multi-view graph convolutional network for identifying spatial domains with attention mechanism. Brief Bioinform 2023; 24:bbad262. 10.1093/bib/bbad262. 37466210

[19] Hang X , Huazhu F, Long Y. et al. Unsupervised spatially embedded deep representation of spatial transcriptomics. Genome Med 2024; 16:12. 10.1186/s13073-024-01283-x. 38217035 PMC10790257

[20] Dong K , Zhang S. Deciphering spatial domains from spatially resolved transcriptomics with an adaptive graph attention auto-encoder. Nat Commun 2022; 13:1739. 10.1038/s41467-022-29439-6. 35365632 PMC8976049

[21] Long Y , Ang KS, Li M. et al. Spatially informed clustering, integration, and deconvolution of spatial transcriptomics with GraphST. Nat Commun 2023; 14:1155. 10.1038/s41467-023-36796-3. 36859400 PMC9977836

[22] Shi X , Zhu J, Long Y. et al. Identifying spatial domains of spatially resolved transcriptomics via multi-view graph convolutional networks. Brief Bioinform 2023; 24:bbad278. 10.1093/bib/bbad278. 37544658

[23] Velickovic P , Cucurull G, Casanova A. et al. Graph attention networks. Stat 2018; 1050:4.

[24] Yu N, Zhang D, Zhang W. et al. stGCL: A versatile cross-modality fusion method based on multi-modal graph contrastive learning for spatial transcriptomics. bioRxiv 2023. 10.1101/2023.12.10.571025. PMC12924433 41606649

[25] Velickovic P , Fedus W, Hamilton WL. et al. Deep graph infomax. Stat 2018; 1050:21.

[26] Scrucca L , Michael Fop T, Murphy B. et al. mclust 5: clustering, classification and density estimation using gaussian finite mixture models. R J 2016; 8:289. 10.32614/RJ-2016-021. 27818791 PMC5096736

[27] Dong K , Zhang S. Deciphering spatial domains from spatially resolved transcriptomics with an adaptive graph attention auto-encoder. Nat Commun 2022; 13:1739. 10.1038/s41467-022-29439-6. 35365632 PMC8976049

[28] Zhou X , Dong K, Zhang S. Integrating spatial transcriptomics data across different conditions, technologies and developmental stages. Nat Comput Sci 2023; 3:894–906. 10.1038/s43588-023-00528-w. 38177758

[29] Hubert L , Arabie P. Comparing partitions. J Classif 1985; 2:193–218. 10.1007/BF01908075.

[30] McDaid AF , Greene D, Hurley N. Normalized mutual information to evaluate overlapping community finding algorithms arXiv preprint arXiv:1110.2515. 2011. 10.48550/arXiv.1110.2515.

[31] Xiao J , Jianfeng L, Li X. Davies bouldin index based hierarchical initialization k-means. Intell Data Anal 2017; 21:1327–38. 10.3233/IDA-163129.

[32] Saraçli S , Doğan N, Doğan İ. Comparison of hierarchical cluster analysis methods by cophenetic correlation. J Inequal Appl 2013; 2013:2013. 10.1186/1029-242X-2013-203.

[33] Tang M , Kaymaz Y, Logeman BL. et al. Evaluating single-cell cluster stability using the jaccard similarity index. Bioinformatics 2021; 37:2212–4. 10.1093/bioinformatics/btaa956. 33165513 PMC8352506

[34] Alexander Wolf F , Angerer P, Theis FJ. SCANPY: large-scale single-cell gene expression data analysis. Genome Biol 2018; 19:1–5. 29409532 10.1186/s13059-017-1382-0 PMC5802054

[35] Zong Y , Tingyang Y, Wang X. et al. CONST: an interpretable multi-modal contrastive learning framework for spatial transcriptomics. BioRxiv 2022;2022–01. 10.1101/2022.01.14.476408.

[36] Gilmore EC , Herrup K. Cortical development: layers of complexity. Curr Biol 1997; 7:R231–4. 10.1016/S0960-9822(06)00108-4. 9162498

[37] McInnes L, Healy J, Saul N, Großberger L. UMAP: Uniform Manifold Approximation and Projection. J Open Source Softw 2018; 3:861. 10.21105/joss.00861.

[38] Alexander Wolf F , Hamey FK, Plass M. et al. PAGA: graph abstraction reconciles clustering with trajectory inference through a topology preserving map of single cells. Genome Biol 2019; 20:1–9. 30890159 10.1186/s13059-019-1663-x PMC6425583

[39] Chen A , Liao S, Cheng M. et al. Spatiotemporal transcriptomic atlas of mouse organogenesis using DNA nanoball-patterned arrays. Cell 2022; 185:1777–1792.e21. 10.1016/j.cell.2022.04.003. 35512705

[40] Stickels RR , Murray E, Kumar P. et al. Highly sensitive spatial transcriptomics at near-cellular resolution with Slide-seqV2. Nat Biotechnol 2021; 39:313–9. 10.1038/s41587-020-0739-1. 33288904 PMC8606189

[41] Kadowaki K , Sugimoto K, Yamaguchi F. et al. Phosphohippolin expression in the rat central nervous system. Mol Brain Res 2004; 125:105–12. 10.1016/j.molbrainres.2004.03.021. 15193427

[42] Sunkin SM , Ng L, Lau C. et al. Allen brain atlas: an integrated spatio-temporal portal for exploring the central nervous system. Nucleic Acids Res 2012; 41:D996–D1008. 10.1093/nar/gks1042. 23193282 PMC3531093

[43] Waldemer-Streyer RJ , Reyes-Ordoñez A, Kim D. et al. Cxcl14 depletion accelerates skeletal myogenesis by promoting cell cycle withdrawal. NPJ Regen Med 2017; 2:1–10. 10.1038/npjregenmed.2016.17. 28775895 PMC5537738

[44] Uchiyama S , Mizutani K, Suzuki E. et al. Bronchiolar adenoma/ciliated muconodular papillary tumor mixed with adenocarcinoma in situ in the same tumor. Thoracic Cancer 2023; 14:427–31. 10.1111/1759-7714.14784. 36578104 PMC9891859

[45] Zeng H , Shen EH, Hohmann JG. et al. Large-scale cellular-resolution gene profiling in human neocortex reveals species-specific molecular signatures. Cell 2012; 149:483–96. 10.1016/j.cell.2012.02.052. 22500809 PMC3328777

