# Supplementary material for: stHGC: a self-supervised graph representation learning for spatial domain recognition with hybrid graph and spatial regularization
Source: Brief Bioinform. 2024 Dec 22;26(1):bbae666. doi: 10.1093/bib/bbae666 (PMC11663487; doi:10.1093/bib/bbae666)
Supplement: Supplementary_file_bbae666 [file supplementary_file_bbae666.pdf]

Supplementary Information for

stHGC: A Self-Supervised Graph Representation Learning for Spatial Domain Recognition with Hybrid Graph and Spatial Regularization

Runqing Wang[1,2], Qiguo Dai[1,2,*] , Xiaodong Duan[1,2] and Quan Zou[3,*]

[1]College of Computer Science and Engineering, Dalian Minzu University, 116600, Dalian, China

[2]SEAC Key Laboratory of Big Data Applied Technology, Dalian Minzu University, 116600, Dalian, China

[3]Institute of Fundamental and Frontier Sciences, University of Electronic Science and Technology of China, 611730, Chengdu, China

[*]Corresponding author:daiqiguo@dlnu.edu.cn(Q.D.); zouquan@nclab.net (Q.Z.)

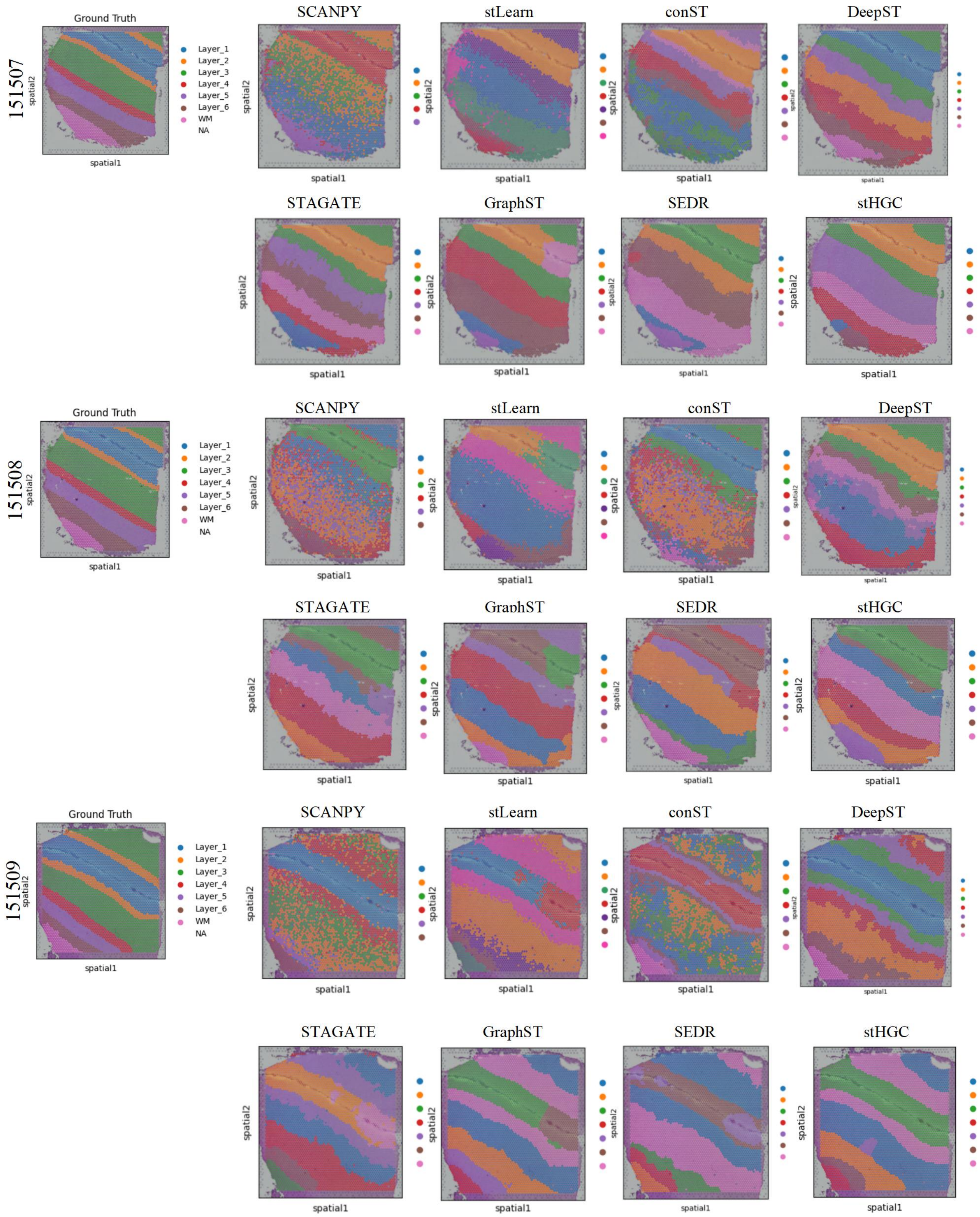

Supplementary Figure 1. The comparison of clustering results by Ground Truth, SCANPY, stLearn, conST, DeepST, STAGATE, GraphST, SEDR, and stHGC in slides 151507, 151508, and 151509 of the DLPFC dataset.

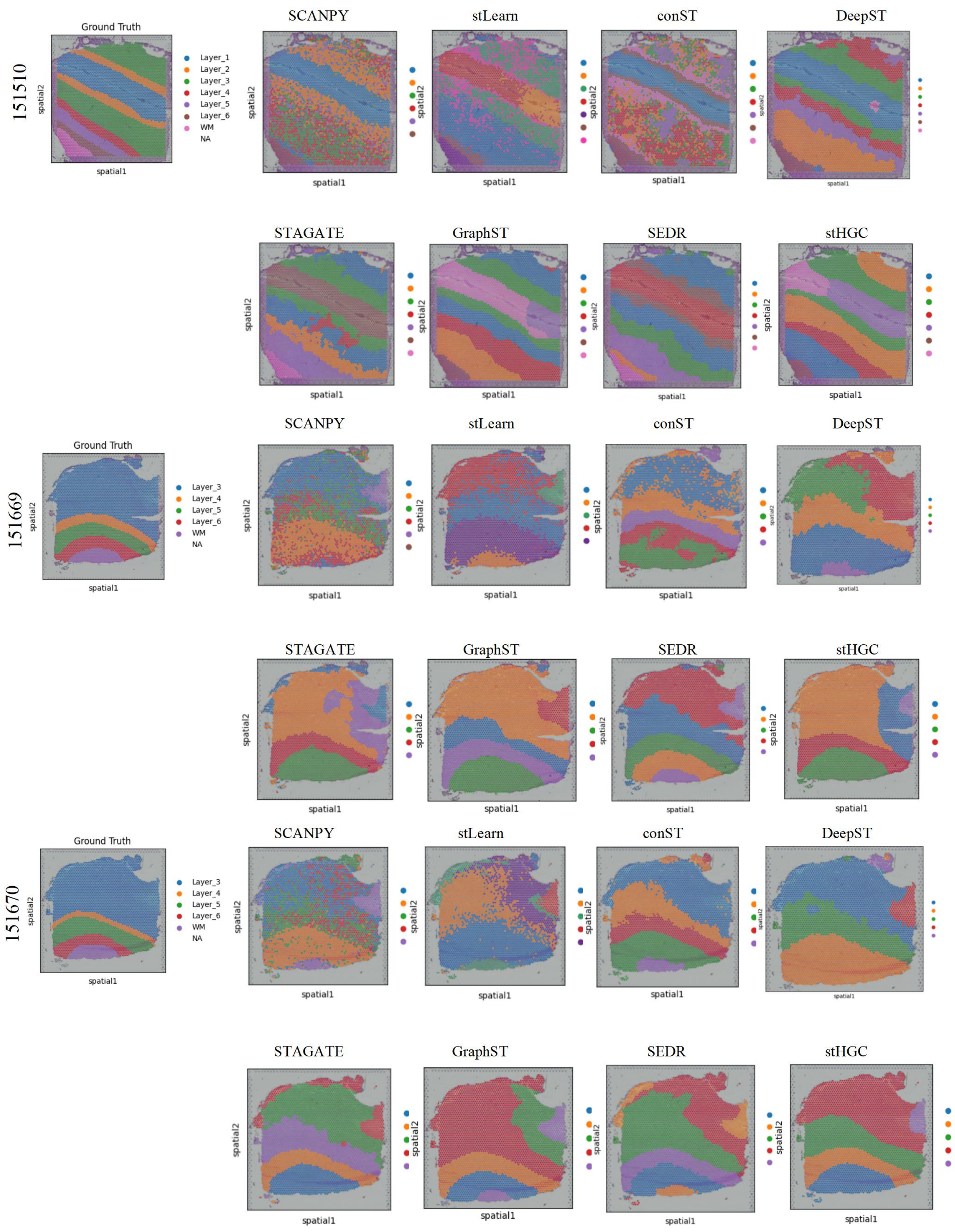

Supplementary Figure 2. The comparison of clustering results by Ground Truth, SCANPY, stLearn, conST, DeepST, STAGATE, GraphST, SEDR, and stHGC in slides 151510, 151669, and 151670 of the DLPFC dataset.

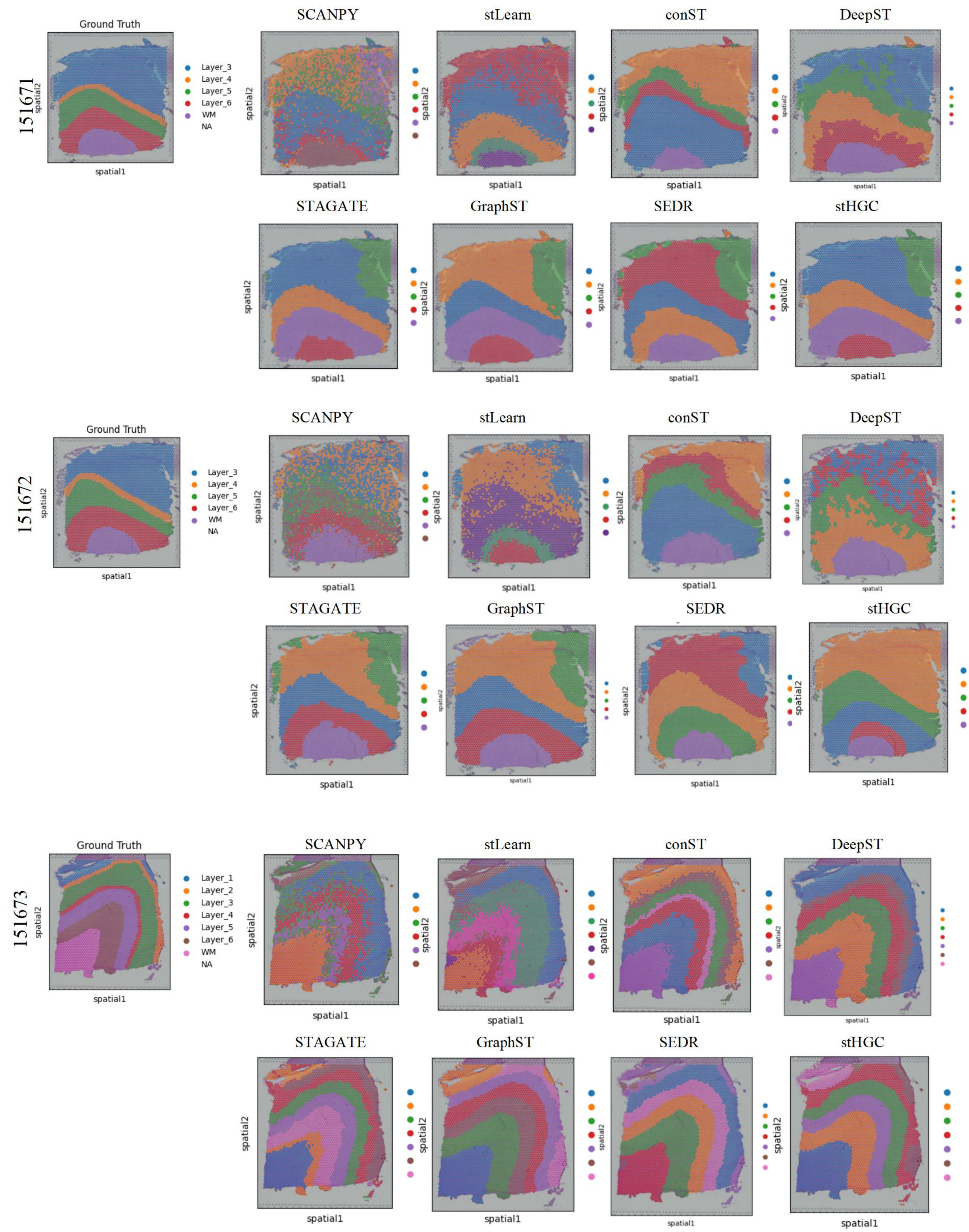

Supplementary Figure 3. The comparison of clustering results by Ground Truth, SCANPY, stLearn, conST, DeepST, STAGATE, GraphST, SEDR, and stHGC in slides 151671, 151672, and 151673 of the DLPFC dataset.

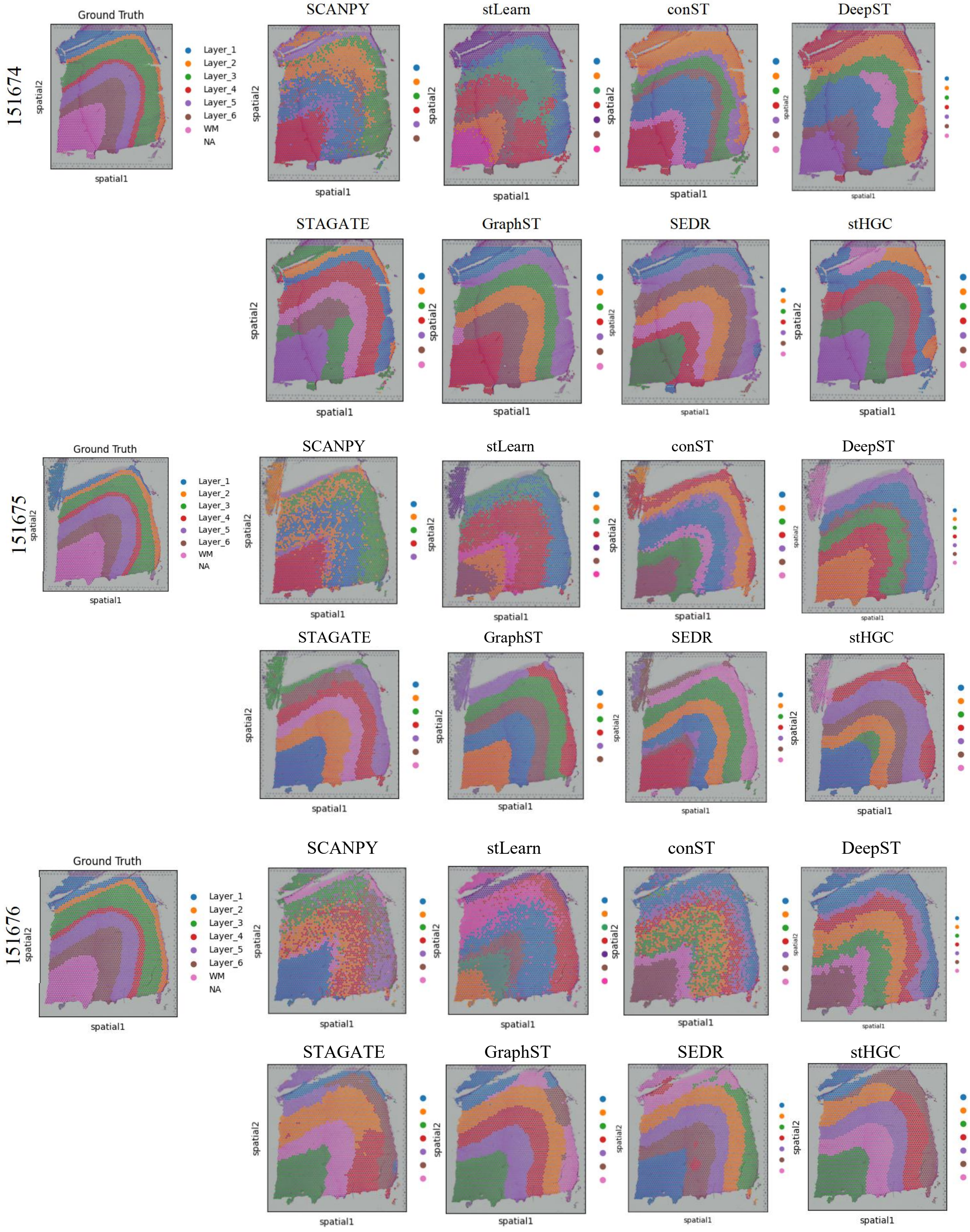

Supplementary Figure 4. The comparison of clustering results by Ground Truth, SCANPY, stLearn, conST, DeepST, STAGATE, GraphST, SEDR, and stHGC in slides 151674, 151675, and 151676 of the DLPFC dataset

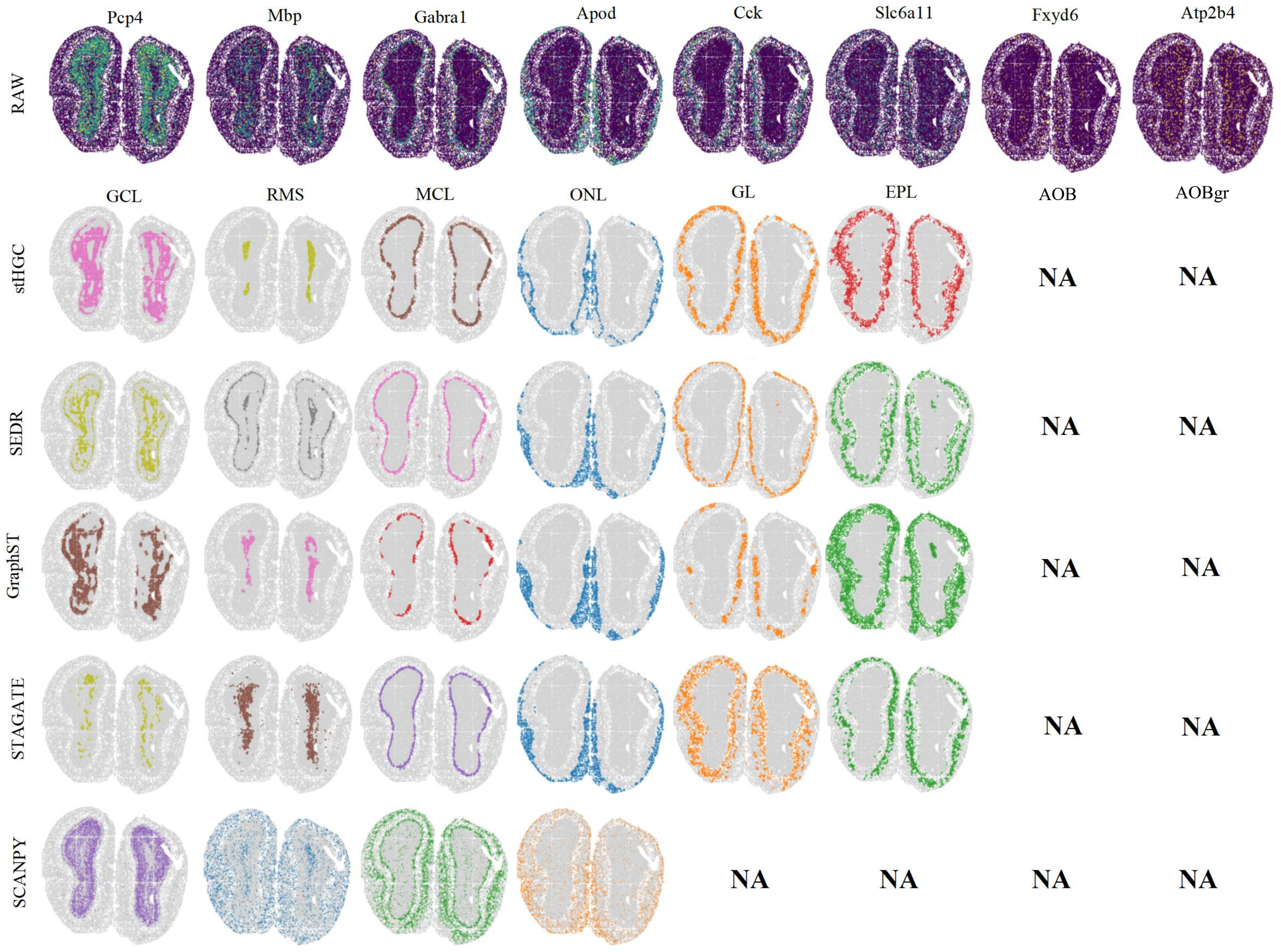

Supplementary Figure 5. The known marker gene expression patterns and the visualizations of each spatial domain identified by stHGC, SEDR, GraphST, STAGATE, and SCANPY in the Mouse Olfactory Bulb (Stereo-seq platform) dataset.

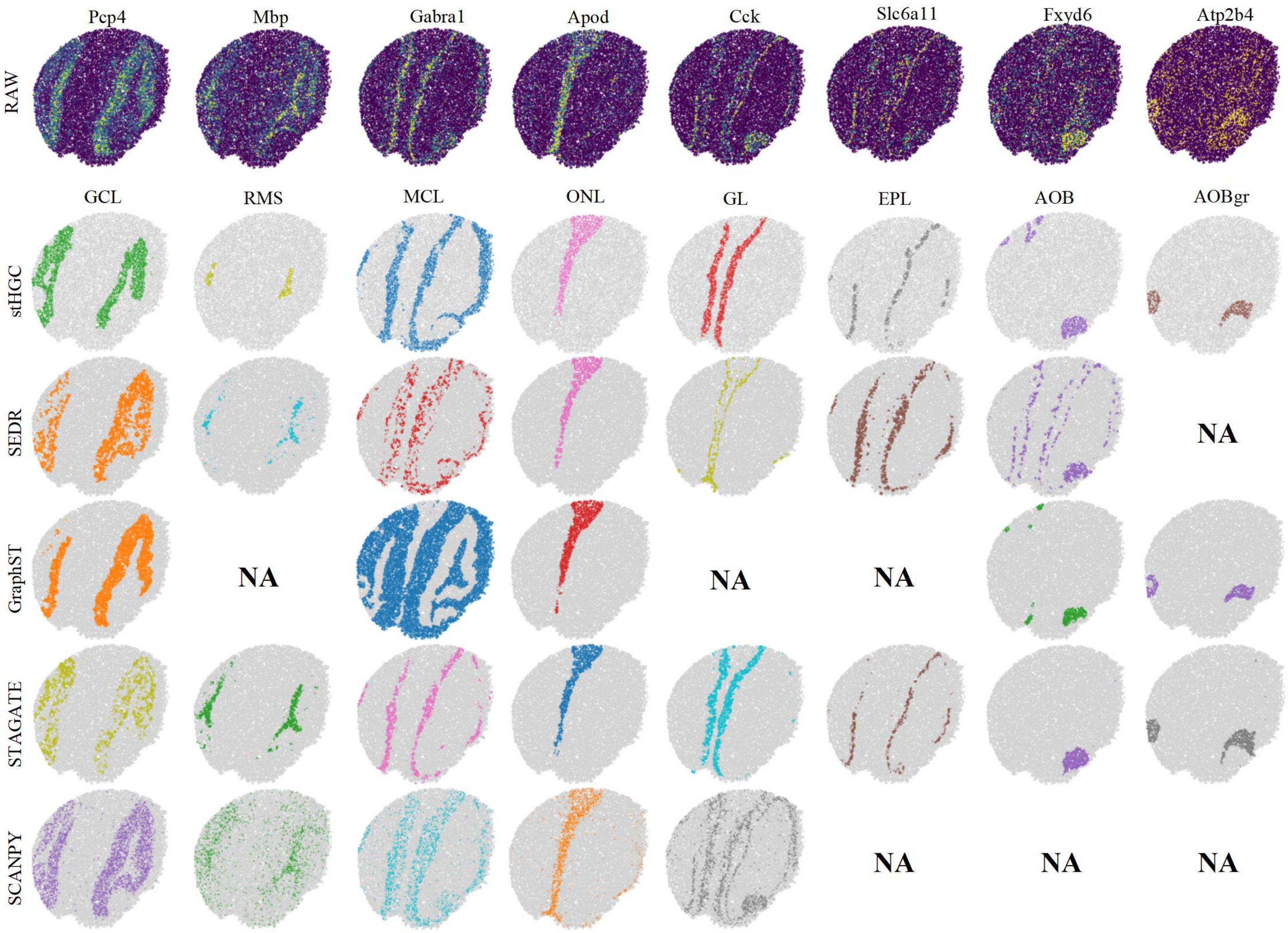

Supplementary Figure 6. The known marker gene expression patterns and the visualizations of each spatial domain identified by stHGC, SEDR, GraphST, STAGATE, and SCANPY in the Mouse Olfactory Bulb (Slide-seqV2 platform) dataset.

Supplementary Table 1: Jaccard Index of stHGC and other state-of-the-art methods on the mouse olfactory bulb dataset (Stereo-seq platform)

Method \ Domain	GCL	RMS	MCL	ONL	GL	EPL	AOB	AOBgr
stHGC	0.28	0.10	**0.22**	0.25	**0.19**	**0.14**	NA	NA
SEDR	0.35	0.09	0.17	0.29	0.04	0.07	NA	NA
GraphST	0.24	0.09	0.14	0.3	0.08	0.12	NA	NA
STAGATE	0.25	**0.11**	0.21	**0.34**	0.05	0.05	NA	NA
SCANPY	**0.49**	0.06	0.04	0.24	NA	NA	NA	NA

Supplementary Table 2: Jaccard Index of stHGC and other state-of-the-art methods on the mouse olfactory bulb dataset (Slide-seqV2 platform)

Method \ Domain	GCL	RMS	MCL	ONL	GL	EPL	AOB	AOBgr
stHGC	0.27	0.05	**0.09**	0.23	0.10	**0.08**	**0.13**	**0.12**
SEDR	0.02	**0.19**	0.03	0.01	0.02	0.02	0.04	NA
GraphST	0.34	NA	0.06	0.21	NA	NA	0.10	0.10
STAGATE	**0.44**	0.12	0.03	0.2	0.10	0.05	**0.13**	0.11
SCANPY	0.34	NA	0.02	**0.27**	**0.37**	NA	NA	NA

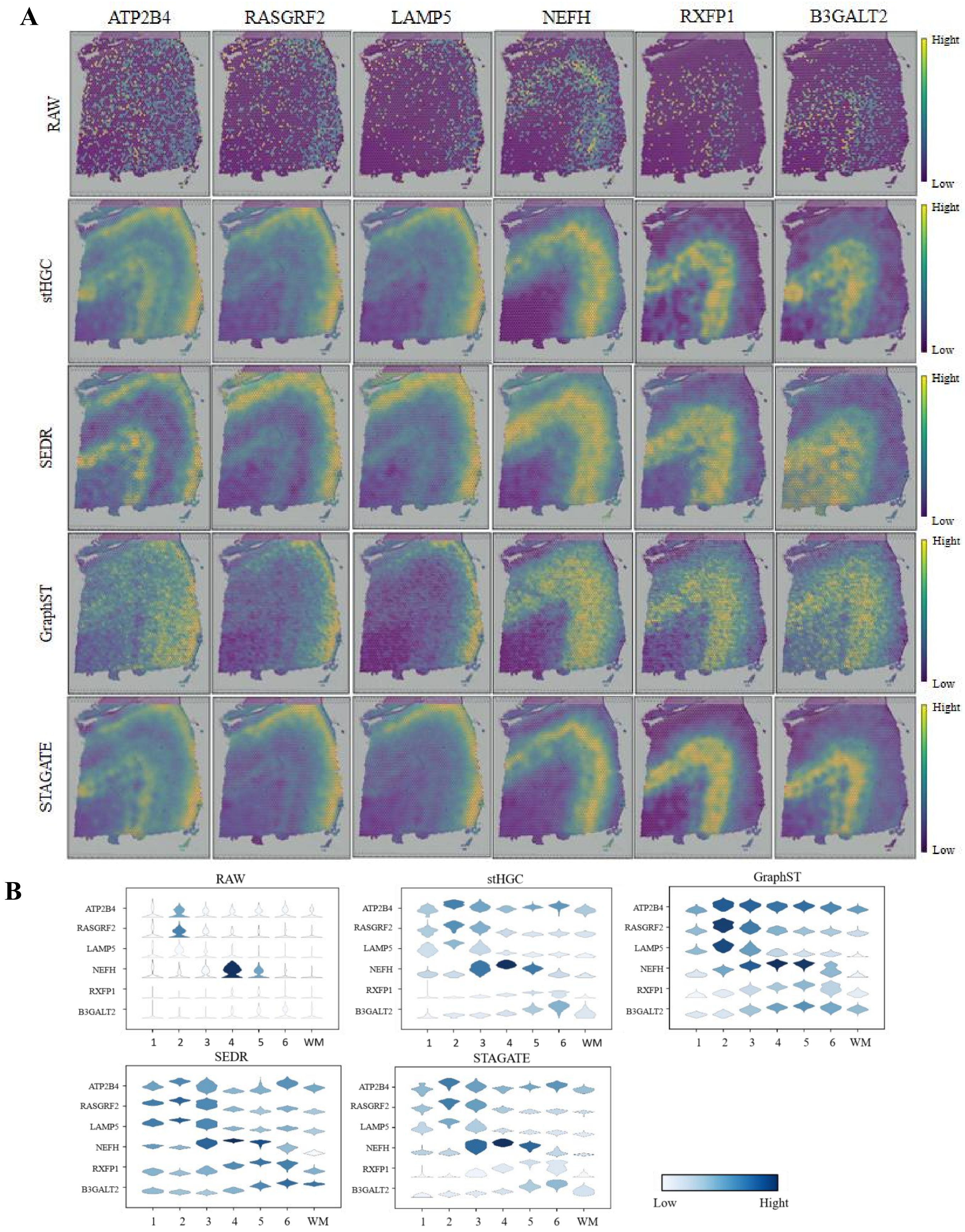

Supplementary Figure 7. Visualization of the original spatial expression and denoised expression by stHGC, SEDR, GraphST, and STAGATE models of six layer marker genes in slice 151673 of the DLPFC. Violin plots of the original expression of layer marker genes and the denoised expression of layer marker genes by stHGC, SEDR, GraphST, and STAGATE models.

Supplementary Table 3: ARI values of stHGC and other state-of-the-art methods on the human DLPFC dataset

Method \ Dataset	151507	151508	151509	151510	151669	151670	151671	151672	151673	151674	151675	151676
SCANPY	0.37	0.33	0.38	0.33	0.26	0.22	0.30	0.37	0.37	0.38	0.36	0.31
stLearn	0.46	0.30	0.42	0.26	0.34	0.19	0.51	0.34	0.36	0.35	0.39	0.36
conST	0.44	0.30	0.32	0.28	0.41	0.44	0.50	0.43	0.55	0.44	0.51	0.33
DeepST	0.53	0.42	0.49	0.46	0.38	0.35	0.55	0.47	0.53	0.46	0.66	0.49
STAGATE	0.59	0.48	0.47	0.53	0.37	0.40	0.59	0.57	0.52	0.59	0.60	0.44
GraphST	0.44	0.49	0.53	0.47	0.6	0.44	0.61	0.62	0.63	0.64	0.62	0.62
SEDR	0.52	0.52	0.39	0.56	0.46	0.34	0.58	0.58	0.63	0.56	0.55	0.60
stHGC	0.56	0.52	0.61	0.46	0.49	0.51	0.63	0.77	0.57	0.6	0.55	0.57

Supplementary Table 4: NMI values of stHGC and other state-of-the-art methods on the human DLPFC dataset

Method \ Dataset	151507	151508	151509	151510	151669	151670	151671	151672	151673	151674	151675	151676
SCANPY	0.43	0.41	0.48	0.40	0.32	0.33	0.43	0.46	0.47	0.48	0.44	0.41
stLearn	0.61	0.52	0.61	0.49	0.51	0.35	0.57	0.47	0.54	0.54	0.56	0.53
conST	0.59	0.38	0.46	0.40	0.56	0.59	0.61	0.63	0.66	0.65	0.64	0.61
DeepST	0.68	0.56	0.64	0.60	0.58	0.50	0.66	0.59	0.67	0.63	0.71	0.65
STAGATE	0.71	0.64	0.64	0.65	0.57	0.57	0.69	0.68	0.65	0.69	0.71	0.69
GraphST	0.64	0.64	0.68	0.64	0.65	0.56	0.72	0.71	0.71	0.73	0.70	0.70
SEDR	0.68	0.64	0.62	0.66	0.61	0.52	0.69	0.69	0.72	0.70	0.68	0.68
stHGC	0.7	0.65	0.67	0.6	0.6	0.62	0.72	0.75	0.69	0.68	0.65	0.67

Supplementary Table 5: Hyperparameter settings for the datasets

Dataset \ Hyperparameter	alpha	beta	lambda	mu	gamma
DLPFC	0.8	0.2	10	10	1
·Olfactory bulb	0.8	0.2	1	4.1	1
Olfactory bulb	0.8	0.2	1	4.1	1
Breast cancer	0.8	0.2	10	10	1
Bronchiolar adenoma	0.8	0.2	10	10	1
